# Long-term consequences of urinary tract infection in childhood: an electronic population-based cohort study in Welsh primary and secondary care

**DOI:** 10.3399/BJGP.2023.0174

**Published:** 2024-05-28

**Authors:** Kathryn Hughes, Rebecca Cannings-John, Hywel Jones, Fiona Lugg-Widger, Tin Man Mandy Lau, Shantini Paranjothy, Nick Francis, Alastair D Hay, Christopher C Butler, Lianna Angel, Judith Van der Voort, Kerenza Hood

**Affiliations:** PRIME Centre Wales, School of Medicine, Cardiff University, Cardiff.; Centre of Research Trials, School of Medicine, Cardiff University, Cardiff.; Division of Population Medicine, School of Medicine, Cardiff University, Cardiff.; Centre of Research Trials, School of Medicine, Cardiff University, Cardiff.; Centre of Research Trials, School of Medicine, Cardiff University, Cardiff.; Aberdeen Centre for Health Data Science, University of Aberdeen, Aberdeen.; Primary Care Research Centre, University of Southampton; Aldermoor Health Centre, Southampton.; Centre for Academic Primary Care, Bristol Medical School, University of Bristol, Bristol.; Nuffield Department of Primary Care Health Sciences, University of Oxford, Oxford.; DECIPHer, School of Social Sciences, Cardiff University, Cardiff.; Noah’s Ark Children’s Hospital for Wales, Cardiff.; Centre for Trials Research, College of Biomedical and Life Sciences, Cardiff University, Cardiff.

**Keywords:** child, preschool, hypertension, infant, kidney failure, chronic, renal insufficiency, chronic, renal scarring, urinary tract infections

## Abstract

**Background:**

Childhood urinary tract infection (UTI) can cause renal scarring, and possibly hypertension, chronic kidney disease (CKD), and end-stage renal failure (ESRF). Previous studies have focused on selected populations, with severe illness or underlying risk factors. The risk for most children with UTI is unclear.

**Aim:**

To examine the association between childhood UTI and outcomes in an unselected population of children.

**Design and setting:**

A retrospective population-based cohort study using linked GP, hospital, and microbiology records in Wales, UK.

**Method:**

Participants were all children born in 2005–2009, with follow-up until 31 December 2017. The exposure was microbiologically confirmed UTI before the age of 5 years. The key outcome measures were renal scarring, hypertension, CKD, and ESRF.

**Results:**

In total, 159 201 children were included; 77 524 (48.7%) were female and 7% (*n* = 11 099) had UTI before the age of 5 years. A total of 0.16% (*n* = 245) were diagnosed with renal scarring by the age of 7 years. Odds of renal scarring were higher in children by age 7 years with UTI (1.24%; adjusted odds ratio 4.60 [95% confidence interval [CI] = 3.33 to 6.35]). Mean follow-up was 9.53 years. Adjusted hazard ratios were: 1.44 (95% CI = 0.84 to 2.46) for hypertension; 1.67 (95% CI = 0.85 to 3.31) for CKD; and 1.16 (95% CI = 0.56 to 2.37) for ESRF.

**Conclusion:**

The prevalence of renal scarring in an unselected population of children with UTI is low. Without underlying risk factors, UTI is not associated with CKD, hypertension, or ESRF by the age of 10 years. Further research with systematic scanning of children’s kidneys, including those with less severe UTI and without UTI, is needed to increase the certainty of these results, as most children are not scanned. Longer follow-up is needed to establish if UTI, without additional risk factors, is associated with hypertension, CKD, or ESRF later in life.

## Introduction

Urinary tract infection (UTI) is a common cause of serious illness and hospital admission in children.^1^^,^^2^ Childhood UTIs can also cause renal scarring and has been associated with long-term complications such as hypertension, pre-eclampsia, and renal failure.^3^^–^6 However, the evidence for this association is weak and has been questioned.^2^^,^^7^^–^10 In addition, the risk of renal scarring in children with less severe UTI or without underlying risk factors is unclear. This needs to be clarified as it informs the correct approach to urine sampling and diagnosis of UTI in children.

A systematic review, published in 2010, found that the prevalence of renal scarring following first childhood febrile UTI was 15%.^5^ Another systematic review, in 2017, examining antibiotic prophylaxis for recurrent UTI, found renal scarring in 5.7% of children.^11^ The studies in these reviews, and those on which much of the current practice is based, were generally conducted in secondary care, where children tend to have serious illness, or in selected populations where a high proportion of children have other risk factors, such as vesicoureteral reflux disease (VUR).

One study included recruitment from primary care and reported that, of 143 children with UTI and without VUR, 5.6% had renal scarring.^12^ However, only 19.7% of eligible children were enrolled in the study suggesting significant selection bias.^12^ Studies examining risk factors for renal scarring, including a meta-analysis of 1280 children, are also limited to children with febrile UTI, those with serious illness in hospital, and with a high proportion of VUR.^13^^–^18

The risk of renal scarring for the majority of children, not necessarily febrile and without additional risk factors, frequently seen in primary care, is unknown. The long-term follow-up of children with UTI has been highlighted by the National Institute for Health and Care Excellence (NICE) as a research priority.^2^ The importance of understanding the risk of complications following UTI in most children, without underlying congenital abnormalities, has also been highlighted.^8^

**Table:** How this fits in

Studies have estimated the risk of renal scarring following childhood urinary tract infection (UTI) to be 5.6%–15%, but these studies were generally conducted in a hospital with selected populations of children who were seriously ill or with additional risk factors. The risk of renal scarring from less severe UTI, without additional risk factors, and in the majority of children commonly seen in primary care, is unknown. This study has found that childhood UTI, including UTI diagnosed in primary care, is associated with renal scarring, even in the absence of other risk factors such as vesicoureteric reflux, but the rate of diagnosed renal scarring is only around 1%. It has also found that childhood UTI, in the absence of other risk factors, is not associated with chronic kidney disease, hypertension, or end-stage renal failure up to the age of 10 years.

The aim of this study was to determine whether children who have experienced a UTI in childhood (before the age of 5 years), from any setting and of any severity, have worse outcomes compared with children who have not experienced a UTI, across the whole population of Wales.^19^ A secondary objective was to identify factors that were associated with renal scarring in children who have had one or more childhood UTI.

## Method

This was a retrospective observational study using anonymised routinely collected data from the Secure Anonymised Information Linkage (SAIL) Databank with person-level linkage across datasets.^20^^–^22

### Data sources

SAIL is a repository for routinely collected health and population data in Wales.^20^^–^22 All data were accessed via the secure, remote-access SAIL Gateway following Information Governance Review Panel approval.^20^^–^22 Public Health Wales provided a data extract of urine microbiology culture results from all microbiology laboratories in Wales (Datastore) for use in this project.

### Study design and participants

The cohort comprised children born and resident in Wales during their first 5 years of life, and who were <5 years of age between 1999 and 2012. The main cohort for analysis was children born between 1 January 2005 and 31 December 2009 to ensure that the first 5 years of life were covered by the dates when microbiology data were available. The study end date was 28 February 2017.

### Exposure

Diagnosis of UTI cases were based on NHS laboratory results from microbiological culture. These data represented samples, from primary and secondary care, classified as positive or negative by NHS laboratories according to their standard operating procedures. Exposure was at least one microbiologically confirmed UTI before the fifth birthday.

As exposure (to UTI) can change over the study period, exposure status was taken at the time of outcome or at age 5 years where no outcome was recorded before this time.

### Outcomes

The primary outcome measure was renal scarring identified in the medical records. There was no single ICD-10 code for renal scarring, so, following discussion with the medical coding department and consultant paediatric nephrologist (a member of the research team), ICD-10 codes were included where renal scarring or chronic pyelonephritis were specified and where coders indicated renal scarring may be coded (N11.0, N11.1, N13.7, N28.8). The secondary outcomes were hypertension, chronic kidney disease (CKD), end-stage renal failure (ESRF), hospital admissions, GP consultations, antibiotic prescriptions, microbiologically confirmed UTI aged 5–7, dysfunctional voiding, renal imaging, renal/urological surgery, and day case admissions. Sources of data and clinical codes to define outcomes, and analysis approach are shown in Supplementary Tables S1 and S2.

GP consultations related to an actual clinician contact rather than planned consultations such as immunisations or medication reviews.^23^

### Risk factors for renal scaring

A directed acyclic graph informed the choice of variables for inclusion in the main analysis (Supplementary Figure S1). The authors considered sex, deprivation, comorbidities, VUR, and other congenital malformations, and perinatal factors as known or possible potential confounders (Supplementary Table S3). As the effects of exposures that were not mediated through these factors were of interest, adjustments for these were made in the models. Clinical codes for congenital malformations and comorbidities are listed in Supplementary Table S4. Comorbidities were treated as time-varying covariates in the same way as exposure. Congenital abnormalities (including VUR) were considered to have been there since birth even if recording of the diagnosis occurred later.

### Statistical analyses

The sample size was based on the outcome of renal scarring in children with microbiologically confirmed UTI and taken as 15% from the 2010 systematic review.^5^ Full details can be found in the protocol article.^19^

Demographical and clinical codes were used to describe baseline characteristics by exposure status to summarise the study population and describe the prevalence of renal scarring by age 5 years and age7 years. Where a lack of evidence of an event occurred in the data then this was taken as ‘no event’ — for example, if no renal scarring code was found then it was assumed that no renal scarring occurred. Missing data were not imputed and a complete case set was used for variables where data were expected but missing, such as age, sex, and ethnicity. Logistic regression was used with discrete time-varying exposure covariates including the exposure variable and risk factors to model the odds of renal scarring in the first 5 and 7 years of life. Odds ratios (ORs) were estimated with 95% confidence intervals (CIs) for exposure to UTI, adjusting for risk factors. Cox regression with discrete time-varying exposure covariates was used to model time to first renal scarring admission. Models were censored at date of death, migration, or end of study. Any deaths that could modify the chance of renal scarring were taken into account by running a competing risks model. Adjusted HRs (aHR) with 95% CI for exposure to UTI were estimated for time-to-event models.

Secondary outcomes were analysed using a combination of logistic; Poisson, or negative binomial regression; or time-to-event models, depending on the nature of data and were pre-specified (Supplementary Table S1). Poisson or negative binomial regression models were used where the outcome was a count of events, and parameter estimates reported as adjusted incident risk ratios (aIRRs) alongside 95% CIs.

Several sensitivity analyses were performed. Because of uncertainty on whether renal scarring codes were sufficiently sensitive to pick up all cases, the primary outcome was expanded to include renal pathology codes (Supplementary Table S5). Any additional renal scarring diagnoses were determined using these data (Supplementary Table S5) in children linked to GP data. Two effect modifiers were pre-specified for subgroup analyses for the primary outcome: sex of child and presence of any renal/urological congenital anomalies. Risk factors for renal scarring in children with at least one childhood UTI were also identified.

### Post-hoc analyses

Two post-hoc analyses were performed. The first analysed children with only one UTI separately from those with >1 UTI. A second analysed the association between UTI and renal scarring excluding children with congenital anomalies, as advised by the independent study steering committee.

SPSS (version 26.0) and Stata SE (version 16.0) were used for all statistical analyses.

## Results

**Figure 1.**
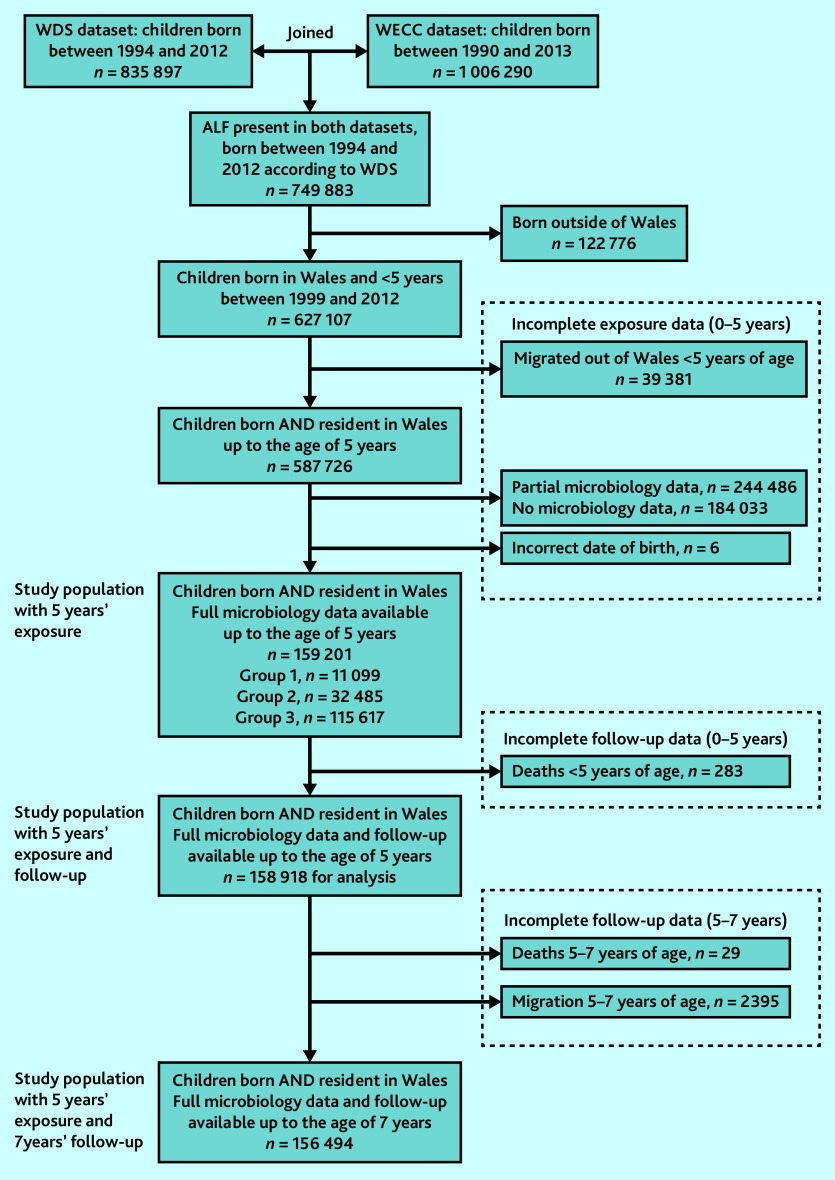
Flow of patients from initial identification in the database through to final cohort. Exposure: Group 1 = at least one microbiologically confirmed UTI; Group 2 = only negative urine samples; Group 3 = no urine samples sent. ALF = anonymised linking field. WDS = Welsh Demographic Survey. WECC = Welsh Cohort for Children. UTI = urinary tract infection.

The study eligible population was 627 107 children born in Wales between 1999 and 2012. After excluding children with an incomplete exposure period before the age of 5 years, 159 201 (25.4%) remained with full exposure data available for all 5 years (Figure 1). Of these, 43 584 (27.4%) children had at least one urine sample analysed between birth and age 5 years. In 32 485 (74.5%) of these children, representing 20.4% of the whole cohort, all of the samples analysed were negative. Of the whole cohort, 11 099 (7.0%) had at least one positive sample (microbiologically confirmed UTI); and 115 617 (72.6%) had no urine samples analysed by the age of 5 years.

The mean follow-up period per child from birth was 9.53 years (standard deviation [SD] 1.54). A total of 158 918 (99.8%) children had at least 5 years’ follow-up, and 156 494 (98.3%) had at least 7 years’ follow-up. The final study cohort was consistent with the population excluded from analysis (Table 1).

**Table 1.** Comparison between study population by exposure group and the excluded population

**Characteristic**	**Group 1 At least one microbiologically confirmed UTI (*n* = 11 099)**	**No UTI (Group 2+3)**		**Group 1+2+3 Final study cohort (*n* = 159 201)**	**Excluded population^a^ (*n* = 428 515)**

		**Group 2 Only negative urine samples (*n* = 32 485)**	**Group 3 No urine samples (*n* = 115 617)**		
**Sex, *n*(%)**					
Male	3359 (30.3)	15 093 (46.5)	63 225 (54.7)	81 677 (51.3)	219 867 (51.3)
Female	7740 (69.7)	17 392 (53.5)	52 392 (45.3)	77 524 (48.7)	208 648 (48.7)

**Deprivation quintile, *n*(%)**					
Missing data	286 (2.6)	908 (2.8)	3254 (2.8)	4448 (2.8)	14 463 (3.4)
Data available	10 813 (97.4)	31 577 (97.2)	112 363 (97.2)	154 753 (97.2)	414 052 (96.6)
1 — Least deprived	2980 (27.6)	7961 (25.2)	29 576 (26.3)	40 517 (26.2)	108 889 (26.3)
2	2425 (22.4)	6698 (21.2)	25 057 (22.3)	34 180 (22.1)	90 340 (21.8)
3	2040 (18.9)	6161 (19.5)	21 008 (18.7)	29 209 (18.9)	78 909 (19.1)
4	1666 (15.4)	5232 (16.6)	19 086 (17.0)	25 984 (16.8)	69 542 (16.8)
5 — Most deprived	1702 (15.7)	5525 (17.5)	17 636 (15.7)	24 863 (16.1)	66 372 (16.0)

**Maternal age at birth, years, *n*(%)**					
Missing data	5 (0.05)	11 (0.03)	50 (0.04)	66 (0.04)	927 (0.2)
Data available	11 094 (99.95)	32 474 (99.97)	115 567 (99.96)	159 135 (99.96)	427 588 (99.78)
≤24	3687 (33.2)	10 384 (32.0)	36 182 (31.3)	50 253 (31.6)	132 425 (31.0)
25–29	3074 (27.7)	9021 (27.8)	30 722 (26.6)	42 817 (26.9)	126 987 (29.7)
30–34	2601 (23.4)	8018 (24.7)	28 755 (24.9)	39 374 (24.7)	110 724 (25.9)
≥35	1732 (15.6)	5051 (15.6)	19 908 (17.2)	26 691 (16.8)	57 452 (13.4)

**Birth weight, grams, *n*(%)**					
Missing data	28 (0.3)	61 (0.2)	128 (0.1)	217 (0.1)	535 (0.1)
Data available	11 071 (99.7)	32 424 (99.8)	115 489 (99.9)	158 984 (99.9)	427 980 (99.9)
Low (<2500)	1065 (9.6)	2616 (8.1)	7090 (6.1)	10 771 (6.8)	28 665 (6.7)
Normal (2500 to <4500)	8877 (80.2)	26 267 (81.0)	94 280 (81.6)	129 424 (81.4)	348 890 (81.5)
High (4000 to <7000)	1129 (10.2)	3541 (10.9)	14 119 (12.2)	18 789 (11.8)	50 425 (11.8)

**Gestational age at birth, weeks, *n*(%)**					
Missing data	81 (0.7)	225 (0.7)	1182 (1.0)	1488 (0.9)	19 565 (4.6)
Data available	11 018 (99.3)	32 260 (99.3)	114 435 (99.0)	157 713 (99.1)	408 950 (95.4)
<33	384 (3.5)	760 (2.4)	1255 (1.1)	2399 (1.5)	6409 (1.6)
33 to <37	700 (6.4)	2085 (6.5)	6042 (5.3)	8827 (5.6)	23 547 (5.8)
37 to 43	9934 (90.2)	29 415 (91.2)	107 138 (93.6)	146 487 (92.9)	378 994 (92.7)

**Ever breastfed,^b^ *n*(%)**					
Missing data	736 (6.6)	1739 (5.4)	7755 (6.7)	10 230 (6.4)	148 445 (34.6)
Data available	10 363 (93.4)	30 746 (94.6)	107 862 (93.3)	148 971 (93.6)	280 070 (65.4)
No	4555 (44.0)	12 667 (41.2)	47 378 (43.9)	64 600 (43.4)	127 851 (45.6)
Yes	5808 (56.0)	18 079 (58.8)	60 484 (56.1)	84 371 (56.6)	152 219 (54.4)

a *Partial (*n *= 244 486) or no (*n *= 184 033) microbiology data, incorrect date of birth (*n *= 6).*

b *At birth or at 6–8 weeks post-partum. UTI = urinary tract infection.*

### Renal scarring

The prevalence of diagnosed renal scarring by age 5 years was 0.13% (*n* = 208), and by age 7 years was 0.16% (*n* = 245). In children with at least one UTI, the prevalence of diagnosed renal scarring by age 5 years was 0.99% (*n* = 109/11 023) and by age 7 years was 1.24% (*n* = 135/10 875) (Table 2). The majority of those with UTI and renal scarring had also been diagnosed with VUR (*n* = 91/109, 83%) (Supplementary Table S10). The association between a UTI and a subsequent diagnosis of renal scarring up to age 5 years was evident (adjusted OR [aOR] 4.03 [95% CI = 2.81 to 5.79]; Table 2). Similar associations were found with renal scarring up to 7 years of age (Table 2). When time to diagnosed renal scarring was examined, the aOR was 2.76 (95% CI = 2.07 to 3.68). A competing risks model produced similar results. Utilising all 159 201 children in the analysis, when time to renal scarring was examined (or in those who did not have renal scarring, time to death, migration, or end of study), the crude hazard ratio (HR) was 16.34 (95% CI = 12.93 to 20.66) and after adjustment for the same confounders reduced to 2.76 (95% CI = 2.07 to 3.68). Using a competing risks model, where the competing risk was death, the crude sub-HR was found to be similar (16.31 [95% CI = 12.91 to 20.62]).

**Table 2.** Renal scarring diagnosis to age of 5 and 7 years by exposure group, *N* = 158 918 and *N* = 156 494, respectively

	**No renal scarring (*n* = 158 710; 99.87%)**	**Renal scarring (*n* = 208; 0.13%)**	**Crude OR (95% CI)**	**Adjusted^a^ OR (95% CI)**
**Analysis to age 5 years**				
No UTI, *n* (%) (*N* = 147 895)	147 796 (99.93)	99 (0.07)	Ref	Ref
At least one UTI, *n* (%) (*N* = 11 023)	10 914 (99.01)	109 (0.99)	14.91 (11.35 to 19.59)	4.03 (2.81 to 5.79)
	**No renal scarring (*n* = 156 249; 99.84%)**	**Renal scarring (*n* = 245; 0.16%)**	**Crude OR (95% CI)**	**Adjusted^b^ OR (95% CI)**
**Analysis to age 7 years**				
No UTI, *n* (%) (*N* = 145 619)	145 509 (99.92)	110 (0.08)	Ref	Ref
At least one UTI, *n* (%) (*N* = 10 875)	10 740 (98.76)	135 (1.24)	16.63 (12.92 to 21.40)	4.60 (3.33 to 6.35)

a *Adjusted for sex, comorbidities, known congenital anomalies, and vesicoureteral reflux (VUR).*

b *Adjusted for comorbidities and vesicoureteral reflux (VUR). OR = odds ratio. Ref = reference. UTI = urinary tract infection.*

### Post-hoc analysis

Children with only one UTI did not have a higher odds of renal scarring diagnosis than those without UTI (aOR 1.34 [95% CI = 0.91 to 1.98]) whereas children with >1 UTI did have a higher odds of renal scarring (aOR 7.09 [4.39 to 11.45]; Supplementary Table S6). The effect of UTI on renal scarring in children without any congenital anomalies, was reduced but still observed (aOR = 2.18 (1.49 to 2.87); Supplementary Table S7).

### Sensitivity analyses

#### Renal pathology

Using any renal pathology codes showed a stronger relationship with UTI than renal scarring codes alone (aOR 5.68 [95% CI = 4.47 to 7.21]) (Supplementary Table S8).

#### GP diagnosis of renal scarring

There were 118 167 (74.2%) children with GP data available in SAIL; however, only 25 were additionally picked up in GP records and so additional analyses were not performed.

#### Subgroup analyses

There was little evidence to show that the effect of UTI was different in males compared with females (interaction term *P* = 0.057) (Supplementary Table S9a).

Children with a renal/urological congenital anomaly were a rare subgroup, but had a much higher rate of renal scarring compared with those with no congenital anomalies (Supplementary Table S9b). There was little evidence to show that the effect of UTI differed between groups (interaction term *P* = 0.068).

### Secondary outcomes

Renal/urological surgery, dysfunctional voiding, experiencing further microbiologically confirmed UTI (age 5 to 7 years), receiving at least one antibiotic, and day case admissions were all associated with UTI (Table 3). After adjustment, there was no association between UTI and hypertension, CKD, or ESRF up to age 5 years or when all available data were used (up to average age of 10 years; Tables 3 and 4). Adjusted hazard ratios were: 1.44 (95% CI = 0.84 to 2.46) for hypertension; 1.67 (95% CI = 0.85 to 3.31) for CKD; and 1.16 (95% CI = 0.56 to 2.37) for ESRF (Table 3).

**Table 3.** Secondary outcomes

**Statistical analysis**	**Hypertension**	**Chronic kidney disease**	**End-stage renal failure**	**Hospital admissions**	**GP consultations**	**Renal/urological surgery**
**Children with a 5 year follow up *N*^a^**	**158 930**	**158 918**	**158 923**	**159 136**	**132 423**	**158 921**
** Children with an event, *n*(%)**						
Negative/no sample^b^	61/147 890 (0.04)	33/147 882 (0.02)	44/147 880 (0.03)	89 938/153 610 (58.6)	119 396/123 522 (96.7)	107/147 910 (0.07)
UTI^b^	15/11 040 (0.14)	13/11 036 (0.12)	7/11 043 (0.06)	3106/5477 (56.7)	8603/8901 (96.7)	73/11 011 (0.66)
Missing	0	0	0	49^c^ (0.03%)	298	0
Crude OR (95% CI), *P*-value	3.30 (1.87 to 5.80), <0.001	5.28 (2.78 to 10.04), <0.001	2.13 (0.96 to 4.73),0.063	0.93 (0.88 to 0.98), 0.007	1.03 (0.92 to 1.16), 0.603	—
Adjusted^d^ OR (95% CI)	1.84 (0.95 to 3.58), 0.072	0.70 (0.29 to 1.72), 0.442	0.92 (0.36 to 2.34), 0.855	0.97 (0.91 to 1.02), 0.239	1.01 (0.90 to 1.14), 0.838	—

**Count of events median (25th to 75th centile)**						
Negative/no sample^b^	—	—	—	2 (1 to 3)	19 (12 to 29)	—
UTI^b^	—	—	—	2 (1 to 3)	27 (17 to 40)	—
CrudeIRR^e^ (95% CI), *P*-value	—	—	—	1.06 (1.02 to 1.10), 0.003	1.40 (1.38 to 1.42), <0.001	—
Adjusted^d^ IRR (95% CI), *P*-value	—	—	—	1.04 (1.00 to 1.08), 0.047	1.39 (1.37 to 1.42), <0.001	—

**Time to first event, years, *N* = 159 201**						
Crude HR (95% CI), *P*-value	3.14 (1.97 to 5.00), <0.001	7.70 (4.61 to 12.87), <0.001	3.83 (2.20 to 6.67), <0.001	0.94 (0.90 to 0.97), <0.001	—	8.95 (6.91 to 11.61), <0.001
CrudeSHR^f^ (95% CI), *P*-value	3.13 (1.97 to 4.99), <0.001	7.69 (4.60 to 12.85), <0.001	3.82 (2.19 to 6.66), <0.001	0.94 (0.91 to 0.97), <0.001	—	8.94 (6.90 to 11.58), <0.001
Adjusted^d^ HR (95% CI), *P*-value	1.44 (0.84 to 2.46), 0.189	1.67 (0.85 to 3.31), 0.140	1.16 (0.56 to 2.37), 0.693	0.96 (0.93 to 0.997), 0.034	—	1.40 (1.00 to 1.96), 0.050

a N *excludes children who die before the age of 5 years unless they have an outcome event, for example, hospital admission).*

b *Exposure taken at end of follow-up period (5 or 7 years) or at time of outcome.*

c *No exposure group since date of admission to hospital was before date of birth.*

d *Adjusted for sex, Index of Multiple Deprivation quintile, birth weight, gestational age (weeks), maternal age (years), ever breastfed, known or possible congenital anomalies and time-varying risk factors: comorbidities (diabetes, malignancies, circumcision, renal surgery, immunosuppression), and any vesicoureteral reflux (VUR). Renal surgery outcome excluded renal surgery from comorbidities.*

e *Negative binomial regression model.*

f *SHR estimate came from a competing risk (CR) analysis where the competing risk was death. HR = hazard ratio. IRR = incidence risk ratio. OR = odds ratio. SHR = subdistribution hazard ratio. UTI = urinary tract infection.*

**Table 4.** Secondary outcomes

**Outcome**	**Renal imaging in general practice**	**Dysfunctional voiding**	**Microbiologically confirmed UTI (5–7 years’ follow-up)**	**Antibiotics**	**Day cases**
**Children with a 5-year follow-up, *N* ^a^**	**132 721**	**132 721**	**156 494^b^**	**132 721**	**158 947**
**Children with an event, *n*(%)**					
Negative/no sample^c^	500/123 589 (0.40)	918/123 560 (0.74)	1746/145 593 (1.2)	101 322/123 522 (82.0)	11 227/148 338 (7.57)
UTI^c^	446/9132 (4.89)	127/9161 (1.39)	686/10 901 (6.3)	8267/9199 (89.9)	1191/10 605 (11.23)
Missing	0	0	0	0	4 (0.003)
Crude OR (95% CI)	12.64 (11.11 to 14.39), <0.001	1.88 (1.56 to 2.26), <0.001	5.53 (5.05 to 6.06), <0.001	1.94 (1.81 to 2.08), <0.001	1.55 (1.45 to 1.65), <0.001
Adjusted^d^ OR (95% CI)	11.80 (10.27 to 13.57), <0.001	1.78 (1.46 to 2.16), <0.001	3.96 (3.59 to 4.37), <0.001	1.92 (1.79 to 2.07), <0.001	1.43 (1.34 to 1.54), <0.001

**Count of events median (25th to 75th centile)**					
Negative/no sample^c^	—	—	—	3 (1 to 6)	—
UTI^c^	—	—	—	5 (2 to 9)	—
Crude IRR^e^ (95% CI), *P*-value	—	—	—	1.49 (1.46 to 1.51), <0.001	—
Adjusted^d^ IRR (95% CI), *P*-value	—	—	—	1.50 (1.47 to 1.53), <0.001	—

a N *excludes children who died before the age of 5 years unless they have an outcome event, for example, hospital admission.*

b *Children with a 7-year follow-up.*

c *Exposure taken at end of follow-up period (5 years) or at time of outcome.*

d *Adjusting for sex, Index of Multiple Deprivation quintile, birth weight, gestational age in weeks, maternal age in years, ever breastfed, known or possible congenital anomalies and time-varying risk factors: comorbidities (diabetes, malignancies, circumcision, renal surgery, immunosuppression), and any vesicoureteral reflux (VUR) (congenital and non-congenital). Note: renal surgery outcome excluded renal surgery from comorbidities.*

e *Negative binomial regression model. IRR = incidence risk ratio. OR = odds ratio. UTI = urinary tract infection.*

Children with UTI experienced more GP consultations than those without (median 27 versus 19 respectively, adjusted incidence risk ratio [aIRR] 1.39 (95% CI = 1.37 to 1.42), Table 3); and received more antibiotic prescriptions (median 5 versus 3 respectively, aIRR 1.50 (1.47 to 1.53), Table 4).

### Exploratory: factors associated with renal scarring in Group 1

In 11 023 children with at least one UTI before the age of 5 years, VUR, having a UTI under the age of 1 year, and ≥3 UTIs before the age of 5 years had the strongest association with renal scarring (Supplementary Table S10).

## Discussion

### Summary

This study describes the outcomes for 159 201 children, including 11 099 with at least one microbiologically confirmed UTI before their fifth birthday, and reflects routine clinical practice across all settings, including primary care. Children who had UTI were more than four times as likely to have a subsequent diagnosis of renal scarring. Children who had at least one UTI before the age of 5 years received more antibiotic prescriptions and were more likely to be diagnosed with dysfunctional voiding and further UTIs (aged 5–7 years) than those without. The strongest predictors of subsequent renal scarring in children experiencing one or more UTI were: VUR, a UTI under the age of 1 year, and ≥3 UTIs under the age of 5 years. After adjustment for risk factors, the authors found no association between microbiologically confirmed UTI and subsequent CKD, hypertension, or ESRF in children up to a mean age of 10 years.

### Strengths and limitations

Though large numbers of children were included in the study, the outcomes of interest were uncommon. Adjustments for many risk factors were made; however, there could be others that were unrecognised. Clinical features such as fever could not be examined using routine data, and the numbers having dimercaptosuccinic acid (DMSA) scans were not coded in hospital data. There was no single ICD-10 code for renal scarring and the validity of using the ICD-10 codes in this study has not been tested. This could have resulted in some renal scarring diagnoses being missed or some incorrectly categorised as renal scarring. The association between VUR and renal scarring may have been overestimated owing to the codes used to define them.

As this is a whole-population study reflecting standard clinical practice, imaging was not systematically performed on all children. Therefore, some cases of renal scarring may not have been identified; and those who were scanned would have been more likely to have had recurrent or more serious UTI, or known risk factors, as current guidance does not recommend scanning children with only one uncomplicated UTI.^2^^,^^24^ Equally, some children may have had undiagnosed VUR, other risk factors, or unsuspected UTI. Though having microbiological confirmation for UTI was a strength of this study, the authors did not explore UTIs that were clinically suspected but not confirmed microbiologically. Studying an unselected population and including all UTIs, diagnosed from any setting, allowed assessment of the impact of multiple UTIs and UTIs of any severity.

### Comparison with existing literature

The present study found that UTI was not associated with hypertension, CKD, or ESRF in the absence of underlying risk factors. Previous evidence is inconclusive; however, the previously held belief that UTI leads to these long-term complications has more recently been questioned.^4^^,^^7^^–^10 The present study was large and included all children with microbiologically confirmed UTI before age 5 years. However, the authors only had follow-up data to an average age of 10 years of age, so could not be sure that UTI is not associated with these outcomes at an older age.

The prevalence of renal scarring in the present study was lower than in previously published studies, though to the authors’ knowledge no other studies have attempted to estimate the prevalence in a large unselected population of children. Previous studies include systematic DMSA scanning but are limited to small, select populations of children with febrile UTIs, usually conducted in hospital, reflecting more severe illness, and a higher proportion with VUR (64%–76%).^5^^,^^11^^,^^25^^–^27 These studies vary widely in reported rates of renal scarring, ranging from 2.8% to 15%.^5^^,^^11^^,^^12^^,^^25^^,^^26^ One study included 50 children with afebrile UTI and found no renal scarring, but the 95% CI was wide (0 to 7).^25^ Not all children with UTI would have been scanned in the present study, so the true prevalence may be higher; however, it had follow-up to an average age of 10 years, covering a long period of time during which a scan could have occurred. The authors also cannot be sure that some children without UTI did not have undiagnosed renal scarring as this would require children without UTI or underlying risk factors to have a DMSA scan. On review of the literature, no studies that included a group of children without UTI or risk factors undergoing DMSA scanning were found.

Very few children in the present study had VUR or underlying risk factors, reflecting more closely the majority of children likely to be seen in primary care (VUR in 0.4% of the study population and in 5.6% of children with at least one UTI).^28^

### Implications for research and practice

Childhood UTI, even in the absence of other risk factors, is associated with renal scarring, suggesting the importance of prompt diagnosis and treatment of UTIs in all children. However, the prevalence of renal scarring appears to be low, and it is not certain that renal scarring leads to poor outcomes in the majority of children, so it is unclear whether urine sampling strategies in primary care need to be modified, and to what extent.^8^

The finding of the present study that UTI is not associated with longer-term complications except in the presence of other risk factors means that it may not be necessary to further image and follow-up children without risk factors, particularly given improvements in antenatal scanning to detect congenital abnormalities. However, as the average follow-up was only 9.58 years in the present study, it is possible that there was insufficient time for complications to have developed, and studies with longer follow-up are needed to confirm the presented findings.

To be sure that renal scarring diagnoses are not being missed in standard clinical practice, and to determine the true relationship between UTI and renal scarring, a large study with systematic DMSA scanning in a low-risk population of children, including those with self-limiting UTI and without UTI, is needed. However, as DMSA scans are invasive tests that do not alter clinical management of the individual in most cases, this may be difficult to justify; and selection bias is likely to remain a significant challenge. Longer follow-up is needed to establish if UTI, without additional risk factors, is associated with hypertension, CKD, or ESRF later in life.

## Data

This study makes use of anonymised data held in the Secure Anonymised Information Linkage (SAIL) system, which is part of the national e-health records research infrastructure for Wales and cannot be shared. The protocol for this article has been published.^19^ Rebecca Cannings-John and Kathryn Hughes had full access to all of the data in the study and take responsibility for the integrity of the data and the accuracy of the data analysis.
